# First person – Fabienne Dreier

**DOI:** 10.1242/bio.062858

**Published:** 2026-09-10

## Abstract

First Person is a series of interviews with the first authors of a selection of papers published in Biology Open, helping researchers promote themselves alongside their papers. Fabienne Dreier is first author on ‘Multiplexed gene expression analysis in dissociated and intact planarian flatworm tissues using *in situ* hybridization chain reaction’, published in BiO. Fabienne is a PhD student in the lab of Jochen C. Rink at Max Planck Institute for Multidisciplinary Sciences, Göttingen, Germany, investigating the biology of regeneration and study the mechanisms of this process in planarian flatworms, which are able to regenerate any lost body part in only a few weeks.

**Figure:**
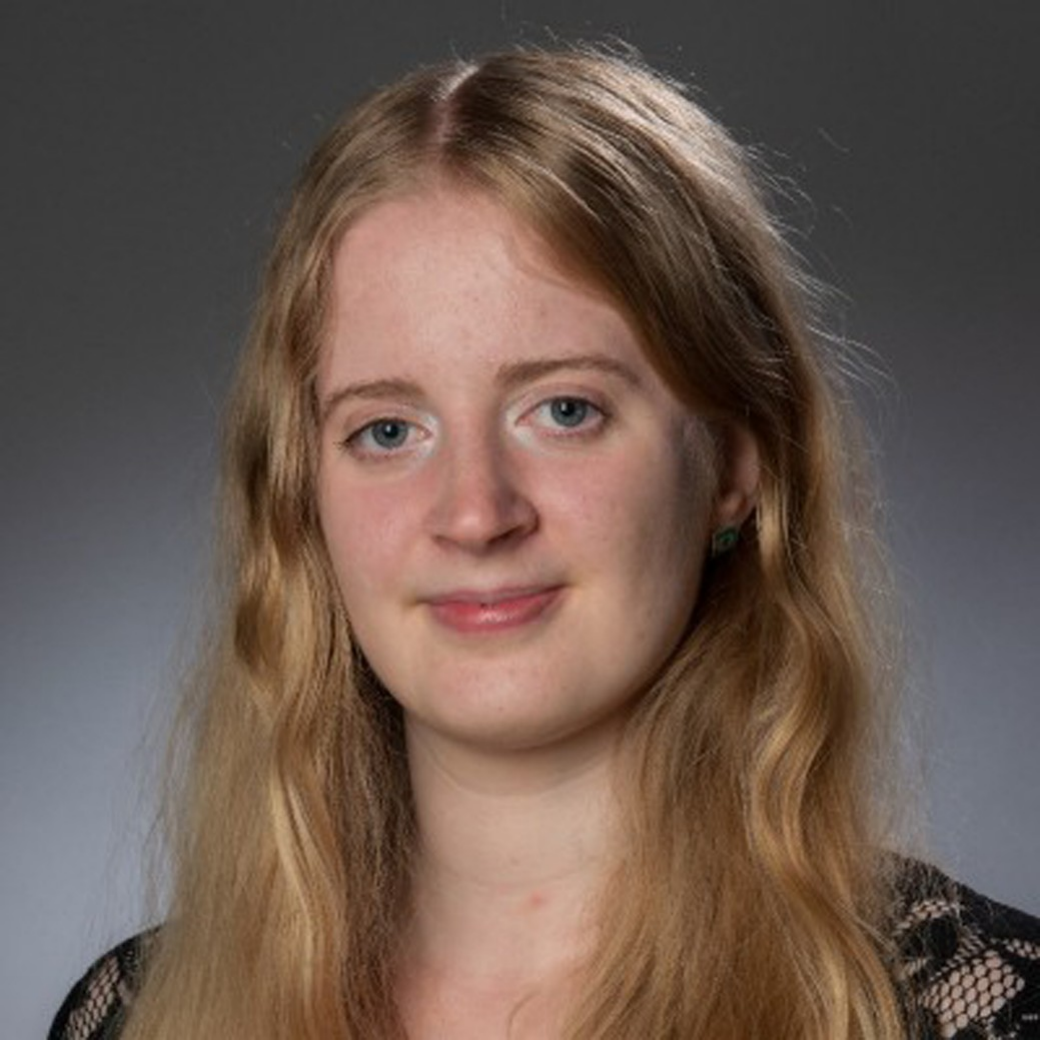
Fabienne Dreier

**Describe your scientific journey and your current research focus**

My scientific journey could be described as ‘topic-hopping’, as I've always had diverse interests. After taking some short courses in biology at the local university during middle and high school, I decided to study biochemistry to get a deeper understanding about how biological processes work. During my Bachelor's and, later, my Master's thesis, I studied the biology of pathogens, specifically the grey mold *Aspergillus fumigatus* and the bacterium *Listeria monocytogenes*. In both cases, it was interesting to learn about how these organisms were able to adapt to treatment with antimicrobial compounds. When stumbling upon planarians after finishing my Master's degree, I became fascinated by the ability of these simple looking worms to heal themselves by regenerating lost body parts. My current research focus is uncovering the mechanisms of mediolateral regeneration. I'm trying to learn how planarians can form a new midline and restore bilateral symmetry in just a tiny piece of tissue taken from nearly anywhere in the body.

**Who or what inspired you to become a scientist?**

Like most biologists, I've always been curious about nature. As I child, I caught the frogs and tadpoles in our pond to look at how they grew. I was the kid who wanted to pet every animal and collect all kinds of leaves, nuts and berries (my parents had to teach me quite early about what I could touch and what I needed to leave be!). During middle and high school, I had the opportunity to learn about marine biology and molecular biology at the local university. These experiences made me aware of how much there is to explore about the living world that surrounds us and led me to pursue a scientific career path.

**How would you explain the main finding of your paper?**

Nearly every cell in every organism has instructions in the form of genes that are critical to maintaining the cell and letting it divide to form more cells. But not every gene is used by every cell. One approach to visualise which genes are ‘on’, or actively expressed in the cells you are studying, is called *in situ* hybridization. This protocol fluorescently tags the RNA products for genes of interest (which basically serve as molecular blueprints) so that the cells glow under the microscope. A recently developed method called hybridisation chain reaction (HCR) allows for the simultaneous marking of several different RNAs. Our paper optimised this protocol for flatworms. We found that soaking the worms in different chemicals and changing the temperature for parts of the protocol results in a brighter signal. The method we developed will make it much easier to observe gene expression in these animals so that we can learn more about their biology, including how they are able to regenerate.

**What are the potential implications of this finding for your field of research?**

The current lack of transgenic approaches in the planarian field has made *in situ* hybridization the method of choice for analyzing gene expression *in vivo*. However, the number of different targets that can be observed at the same time are limited with established protocols. Our HCR approach offers researchers the opportunity to use multiplex experiment setups that can speed up their work by visualising the expression of several genes in the same animal with high sensitivity.

**Figure:**
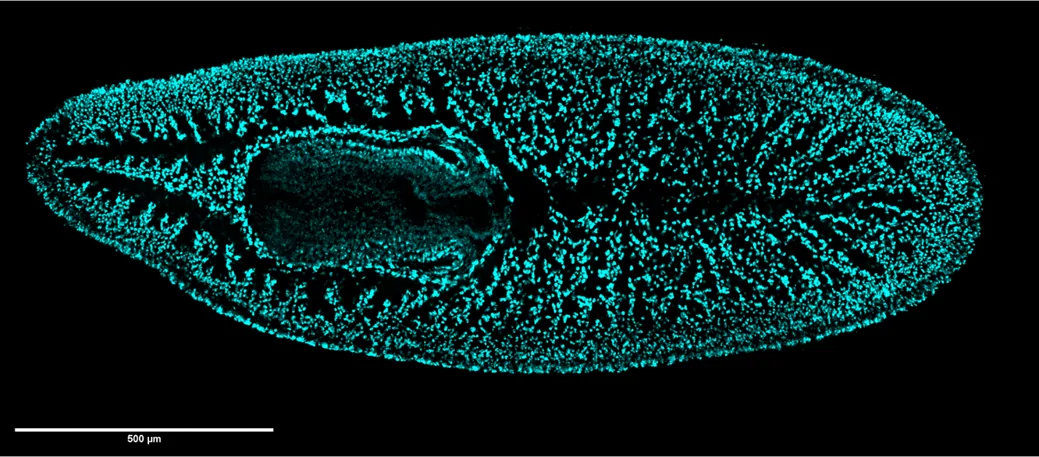
HCR of collagen-positive cells inside a planarian.

**Which part of this research project was the most rewarding?**

The most rewarding part was comparing our final protocol with the one we started with, which was developed for use with other organisms. The improvements were immediately clear under the microscope and became even more obvious with imaging to filter background signal. This was the moment I realised our work made a huge difference for planarian samples and that all my trial-and-error experiments were very much worthwhile.

**What do you enjoy most about being an early-career researcher?**

Learning so many new things. It can be a lot, especially in the beginning, but I notice how each new subject that I dive into changes my scientific perceptions. For example, I recently read a planarian paper that showed a connection between the dorsoventral boundary and patterning of the anteroposterior axis (Maybrun et al., 2025). My work focuses on the mediolateral axis, but this paper is a good reminder that you have to pay attention to more than just your own little area of expertise because there are all kinds of unexplored connections between biological pathways and processes that might influence your research ideas.

**What piece of advice would you give to the next generation of researchers?**

Try again. Things might take longer than expected, the results might not be what you envisioned, or there may be no new findings at all. Evaluate what you have, do some more research about subjects related to your topic, make a new plan, and then try again. Most successful experiments take time and several tries.

**What's next for you?**

Now, I will focus on the biological part of my PhD thesis. Planarians are bilaterally symmetric animals with a complex body plan that includes a pharynx, body wall muscles, two brain lobes with nerve cords, two eyes, and a gut. As mentioned above, they are able to regenerate any of these body parts in just a few weeks. For my project, I am looking at the regeneration of lateral tissue fragments that are created by parasagittal amputation. Those asymmetric tissue pieces remarkably possess the ability to form an entire new body and regain the bilateral symmetry that the worm possessed before the amputation. My current focus is the regeneration of the midline, which is the mirror axis of a bilateral organism. I am studying which genes are involved in which step of *de novo* midline formation. This involves a lot of *in situ* hybridizations using our new HCR protocol. Finally, in a year's time, I will hopefully be writing my thesis and preparing for my defense.

**Why are you working with planarians?**

When I first heard of planarians, I was fascinated by their regenerative abilities. Planarians do not just heal wounds; they restore their entire body plan even when regenerating from small tissue fragments. This raises lots of interesting questions about how they can tell one end of a body axis from the other and how they reestablish their body axes during regeneration. We now have a growing toolkit to try and answer these questions. For example, the HCR protocol we report here makes it much easier to study the genes involved. I'm using it to investigate which genes play a role in the process of regaining bilateral symmetry within lateral tissue fragments.
